# A Symmetric-Actuating Linear Piezoceramic Ultrasonic Motor Capable of Producing a Scissoring Effect

**DOI:** 10.34133/research.0156

**Published:** 2023-06-06

**Authors:** Zhanmiao Li, Xingyu Yi, Rongqi Zhu, Zhonghui Yu, Xiaoting Yuan, MohammadJavad PourhosseiniAsl, Shuxiang Dong

**Affiliations:** ^1^ School of Materials Science and Engineering, Peking University, Beijing 100871, China.; ^2^ College of Mechatronics and Control Engineering, and Institute for Advanced Study, Shenzhen University, Shenzhen 518051, China.

## Abstract

Conventionally, to produce a linear motion, one motor’s stator is employed to drive one runner moving forward or backward. So far, there is almost no report of one electromechanical motor or piezoelectric ultrasonic motor that can directly generate two symmetrical linear motions, while this function is desired for precise scissoring and grasping in the minimally invasive surgery field. Herein, we report a brand-new symmetric-actuating linear piezoceramic ultrasonic motor capable of generating symmetrical linear motions of two outputs directly without additional mechanical transmission mechanisms. The key component of the motor is an (2 × 3) arrayed piezoceramic bar stator operating in the coupled resonant mode of the first longitudinal (*L*_1_) and third bending (*B*_3_) modes, leading to symmetric elliptical vibration trajectories at its two ends. A pair of microsurgical scissors is used as the end-effector, demonstrating a very promising future for high-precision microsurgical operations. The sliders of the prototype show the following features: (a) symmetrical, fast relative moving velocity (~1 m/s) outward or inward simultaneously; (b) high step resolution (40 nm); and (c) high power density (405.4 mW/cm^3^) and high efficiency (22.1%) that are double those of typical piezoceramic ultrasonic motors, indicating the full capacity of symmetric-actuating linear piezoceramic ultrasonic motor working in symmetric operation principle. This work also has enlightening significance for future symmetric-actuating device designs.

## Introduction

Precise scissoring and grasping in minimally invasive surgery have been widely involved in various medical or biomedical application fields such as micro-biopsy of tumor cells [1,2], retinal microsurgery [3], gastrointestinal mucosal surgery [4,5], etc., while the actuating manipulator is the core component in the robot-assisted micro/nano operation technology [6–8]. The scissoring or grasping operation is essentially a symmetrical actuation between two handles of one pair of scissors or a pair of tweezers, in which they relatively move (toward or away from each other) simultaneously. However, the conventional piezoelectric or electromechanical motors/actuators use single or even multiple stators to drive one runner for generating forward or backward movements [9–11]. To produce symmetric actuations and movements, normally, two series-connected or parallel-connected stators are required to respectively drive two runners moving toward or away from each other simultaneously; it is also possible to employ sophisticated transmission mechanisms such as racks and pinions, flexible hinges, etc., to convert one-direction movement of one motor into two opposite movements, but at the cost of the cumbersome structure and very limited stroke [12,13].

Recently, diversified motors are employed in the manipulators, such as pneumatic motors [14], electromagnetic motors [15–17], shape memory alloy motors [18,19], and piezoelectric motors [7,8,20–22]. Because of the superiorities of high resolution, large power density, high integration capability, and self-lock performance when power is off [23,24], piezoelectric motors stand out when compared with other types, rendering them particularly attractive and increasingly adopted. However, there has been a lack of operation mechanism of the symmetrical linear motions, regardless of electromechanical motors or piezoelectric ultrasonic motors. The existing reports mainly take care of the structure, material, and performance improvements of piezomotors on the basis of traditional operation mechanisms [25–27]. Therefore, it is necessary to develop a new driving mechanism to achieve symmetrical high-precision motions with a large movement range of two runners driven by only one miniaturized and integrated piezoelectric stator or actuator.

Currently, widely used piezoelectric actuation structures include (a) the bolt-clamped metal–ceramic structures [28,29], (b) the bulk-type ceramic or bonded metal–ceramic structures [30–32], and (c) co-fired ceramic structures [33–35]. Their actuation performances vary with the characteristics of the structures. The bolt-clamped metal–ceramic motors are mainly used for operations under a strong electric field at the resonant frequency. The bolt-clamped method could effectively avoid the cracking problem of piezoelectric ceramics, but at the cost of complicated structures and large volumes. On the contrary, the co-fired ceramic piezoelectric motors are mainly suitable for working under low voltage at non-resonant frequency, although they have the advantages of miniaturization and integration.

In this work, we designed a bonded metal–ceramic structured piezoelectric bar that contains (2 × 3) arrayed units and operates in the coupled resonant mode of the first longitudinal (*L*_1_) and third bending (*B*_3_) modes to produce two symmetrical elliptical movement trajectories in opposite directions at its two ends. For the first time, a novel symmetric-actuating linear piezoceramic ultrasonic motor (SLPUM) that incorporates one pair of scissors was achieved by using the bar as the piezoelectric stator, which could directly generate two-way symmetrical motion outputs of one pair of scissors without using additional complex transmission mechanisms. Moreover, the presented SLPUM exhibits excellent actuation features such as compact size, fast motion velocity, large power density, high step resolution, wide velocity range, and great controllability, posing a very promising future in the field of microsurgical robots for performing micro/nano manipulation, precise grasping, and scissoring functions.

## Results

### Actuator structure and operation principle

**Fig. 1.**
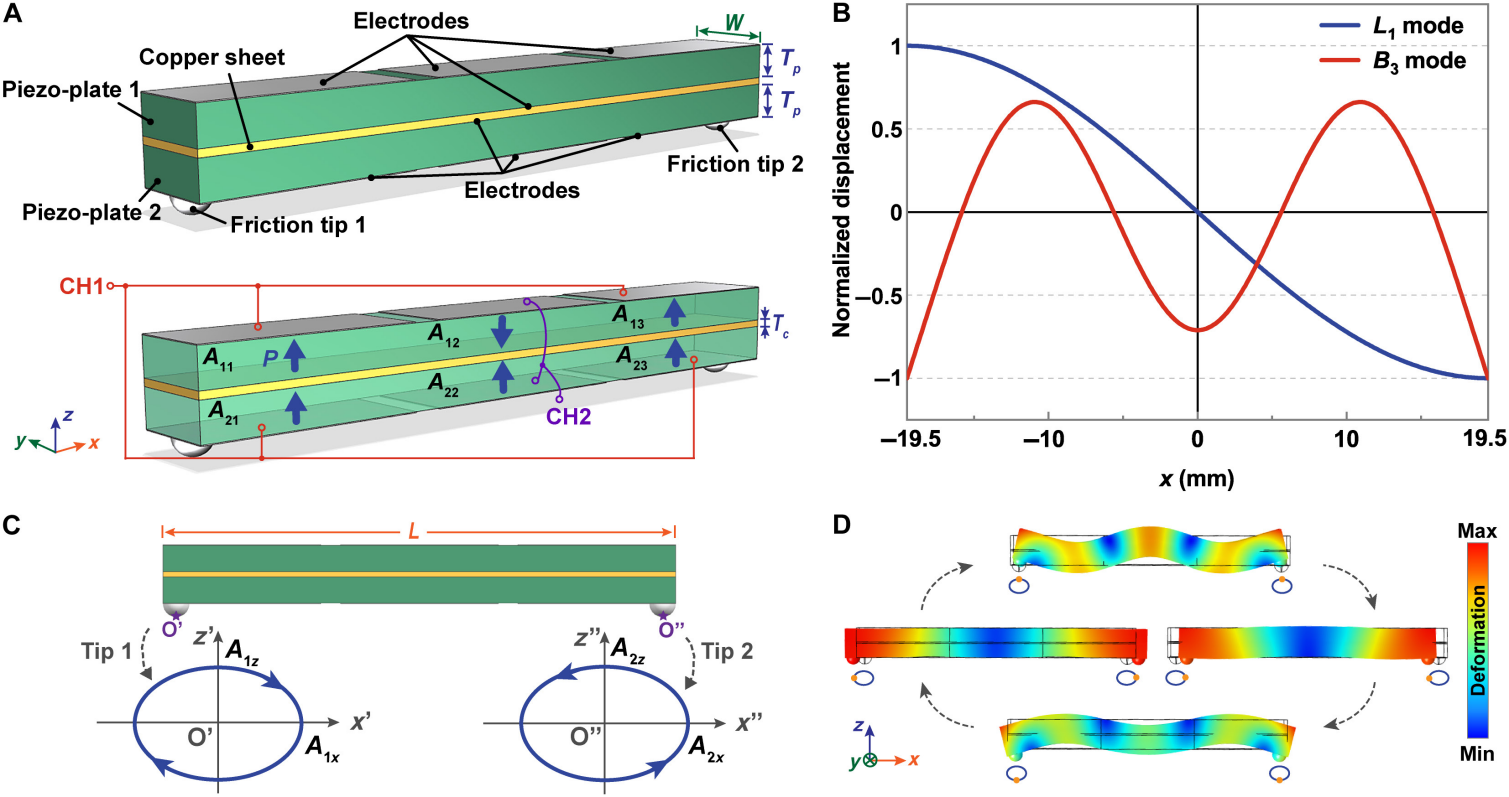
The design and working principle of the piezoelectric stator operating in *L*_1_–*B*_3_ mode. (A) The 3-dimensional model and exciting ways of the proposed piezoelectric stator. (B) Vibration mode shapes of the *L*_1_ mode and *B*_3_ mode obtained from analytical solutions. (C) Motion trajectories of two friction tips in theory. (D) Simulated vibration sequence of the piezoelectric bar stator.

Figure 1A illustrates the 3-dimensional structure of the piezoelectric bar stator operating in *L*_1_–*B*_3_ mode, which is composed of two Pb(Zr,Ti)O_3_ (PZT) ceramic plates, a thin piece of copper sheet, and two friction tips. All components are bonded together by epoxy resin. The silver (Ag) electrodes coated on the top surface of piezo-plate 1 and the bottom surface of piezo-plate 2 are divided into three sections with the same size, while the other two electrodes remain whole for electric ground. With the aid of these divided electrodes applying the desired polarizing and driving electric fields, the piezoelectric bar stator actually forms an (2 × 3) arrayed ordered structure with six piezoceramic strain units named *A_ij_* (*i* = 1, 2; *j* = 1, 2, 3, where *i* and *j* refer to the unit number along the *z*- and *x*-axes), in which *A*_11_, *A*_13_, *A*_21_, *A*_22_, and *A*_23_ are polarized along the positive *z*-direction, whereas *A*_12_ is polarized along the negative *z*-direction, as depicted by the arrows ***P*** in Fig. 1A. According to the principle of maximum strain, the four units at the two ends (*A*_11_, *A*_13_, *A*_21_, and *A*_23_) are used to generate the *B*_3_ mode, and the two units in the middle (*A*_12_ and *A*_22_) are employed to excite the *L*_1_ mode. To realize the best output characteristics, the area of the divided electrodes was almost selected as the maximum value within a reasonable range. Therefore, elliptical movements with reverse directions at the bar’s two ends can be generated by the coupled *L*_1_ mode along the *x*-axis and the *B*_3_ mode along the *z*-axis under two alternating exciting signals (*V*_1_ and *V*_2_). These two voltage signals with the same frequency and a phase difference of 90° or −90° are respectively applied to the electrode input ends named CH1 and CH2. Firstly, we analyze the case where the phase difference between the two signals is 90°, and they satisfy the following relationships:

$$V_{1}=V_{10}e^{\mathrm{jwt}}$$ (1)

$$V_{2}=V_{20}e^{j\left(\mathrm{wt}+\frac{\pi }{2}\right)}$$ (2)

where *V*_10_ and *V*_20_ are the voltage amplitudes, and *ω* is the frequency of the *L*_1_ mode and *B*_3_ mode.

Since the excitation of elliptical trajectories of the friction tips is an important prerequisite for the normal operation of the *L*_1_–*B*_3_ SLPUM, we firstly analyzed the mode vibrations in theory. To simplify the analysis, there are some assumptions: (a) the lateral deformation along the *z*-axis of the piezoceramic bar can be ignored when considering the *L*_1_-mode vibration; (b) when the *B*_3_ mode is generated, the piezoelectric stator can be regarded as a Bernoulli–Euler beam, and the shear strain on its cross-sections and the angular distortion due to shear strain can be reasonably neglected; (c) the effects of parasitic modes and damping can be omitted. Thus, the longitudinal strain *S*_1_ (*S*_1p_ for the piezoceramic plates and *S*_1c_ for the copper sheet) and longitudinal stress *T*_1_ (*T*_1p_ for the piezoceramic plates and *T*_1c_ for the copper sheet) are the major ones, and the corresponding constitutive equations for this piezoelectric stator can be expressed as:

$$S_{1p}=s_{11}^{E}T_{1p}+d_{31}E_{3}$$ (3)

$$D_{3}=d_{31}T_{1p}+\varepsilon_{33}^{T}E_{3}$$ (4)

where *E*_3_ and *D*_3_ are the electric field and dielectric displacement along the *z*-axis; $s_{11}^{E}$, *d*_31_, and $\varepsilon_{33}^{T}$ are short-circuit elastic compliance coefficient, piezoelectric strain coefficient, and stress-free dielectric constant of the piezoceramic plates, respectively.

The constitutive equation for the middle copper sheet is:

$$S_{1c}=s_{11c}T_{1c}$$ (5)

where *s*_11c_ is the elastic compliance coefficient of the copper sheet.

Combining Newton’s second law and elasticity theory [27,36,37], the longitudinal vibration displacement along the *x*-axis *ζ* = *ζ*(*x*, *t*) and the lateral vibration displacement along the *z*-axis *η* = *η*(*x*, *t*) of the beam can be deduced as:

$$\zeta \left(x,t\right)=\left(\mu_{P}V_{20}\right)\zeta_{0}\left(x\right)e^{j\left(\mathrm{wt}+\frac{\pi }{2}\right)}$$ (6)

$$\eta \left(x,t\right)=\left(\varphi_{P}V_{10}\right)\eta_{0}\left(x\right)e^{\mathrm{j\omega t}}$$ (7)

where *μ_P_* and *φ_P_* are the electromechanical conversion factors of the longitudinal vibration and the lateral vibration, respectively, and the expressions of each coefficient are declared in Text S1. To visualize it, the vibration mode shapes for both *L*_1_ and *B*_3_ modes are depicted according to Eqs. 6 and 7, as shown in Fig. 1B. For better driving performances, it is rational to attach two friction tips at two ends of the stator, where *L*_1_ and *B*_3_ modes both have the maximum vibration amplitudes. To avoid vibration displacement loss, one piezoelectric stator needs to be clamped at its node positions, while 2 nodes of the *B*_3_ mode near the middle part of the piezoelectric bar stator are most suitable for clamping to reduce the influence on the vibrations of piezoceramic bar, and the related experiment results can be found in Text S2 and Fig. S1. Meanwhile, the two symmetric-actuating equivalent loads at the two ends of the piezoceramic bar stator is also critical to generate full output power with the minimum clamping (displacement) loss and energy loss.

Thus, the longitudinal (i.e., horizontal) displacement response *ζ*_1_(*t*) and lateral (i.e., vertical) displacement response *η*_1_(*t*) for friction tip 1 can be derived as:

$$\zeta_{1}\left(t\right)=-\left(\mu_{P}V_{20}\right)\zeta_{1}\sin\;\omega t=A_{1x}\sin\;\omega t$$ (8)

$$\eta_{1}\left(t\right)=\left(\varphi_{P}V_{10}\right)\eta_{1}\cos\;\omega t=A_{1z}\cos\;\omega t$$ (9)

where *A*_1*x*_ and *A*_1*z*_ are the vibration amplitudes along the *x*-axis and *z*-axis, respectively. Obviously, the motion trajectory of friction tip 1 is an ellipse whose expression is:

$${\left(\frac{\zeta_{1}\left(t\right)}{A_{1x}}\right)}^{2}+{\left(\frac{\eta_{1}\left(t\right)}{A_{1z}}\right)}^{2}=1$$ (10)

Similarly, friction tip 2 also vibrates in an elliptical trajectory as:

$${\left(\frac{\zeta_{2}\left(t\right)}{A_{2x}}\right)}^{2}+{\left(\frac{\eta_{2}\left(t\right)}{A_{2z}}\right)}^{2}=1$$ (11)

where *ζ*_2_(*t*), *η*_2_(*t*), *A*_2*x*_, and *A*_2*z*_ represent the horizontal displacement, vertical displacement, horizontal vibration amplitude, and vertical vibration amplitude, respectively. It should be noted that the motion trajectories originated from the coupled motion of two perpendicular vibrations generated by *L*_1_ and *B*_3_ modes with the same frequency. The corresponding motion trajectories from Eqs. 10 and 11 are drawn in Fig. 1C. Due to the relationships of *ζ*_1_(*t*) =  − *ζ*_2_(*t*) and *η*_1_(*t*) = *η*_2_(*t*), these two friction tips vibrate in opposite directions, as clearly shown in the operating sequence simulated by using Finite Element Modeling (FEM) with the module of piezoelectric device in COMSOL Multiphysics (see Fig. 1D). Therefore, two runners (or sliders) can be respectively driven by two friction tips in symmetrical directions via friction coupling with only one piezoelectric stator, and the directions of the runners can be reversed by changing the phase difference between CH2 and CH1 from 90° to −90°.

### Characteristics of the *L*_1_–*B*_3_ piezoelectric stator

To realize two symmetric elliptical vibration trajectories at the two friction tips, *L*_1_ and *B*_3_ modes are required to be excited simultaneously in the piezoelectric bar, so the modal simulation was carried out to tune these resonant frequencies to be as close as possible. To balance the electromechanical coupling effect and heat generation problem, the PZT-4 ceramic (provided by HongSheng Acoustics Co., Ltd., China) with the piezoelectric strain coefficients *d*_33_, *d*_31_, and *d*_15_ of 2.8×10^−10^ C/N, −1.0×10^−10^ C/N, and 4.2×10^−10^ C/N was selected as the material of piezo-plates, and the other physical parameters are listed in Table S1. The hemispherical friction tips with a diameter of 2 mm were made of zirconia ceramic.

**Fig. 2.**
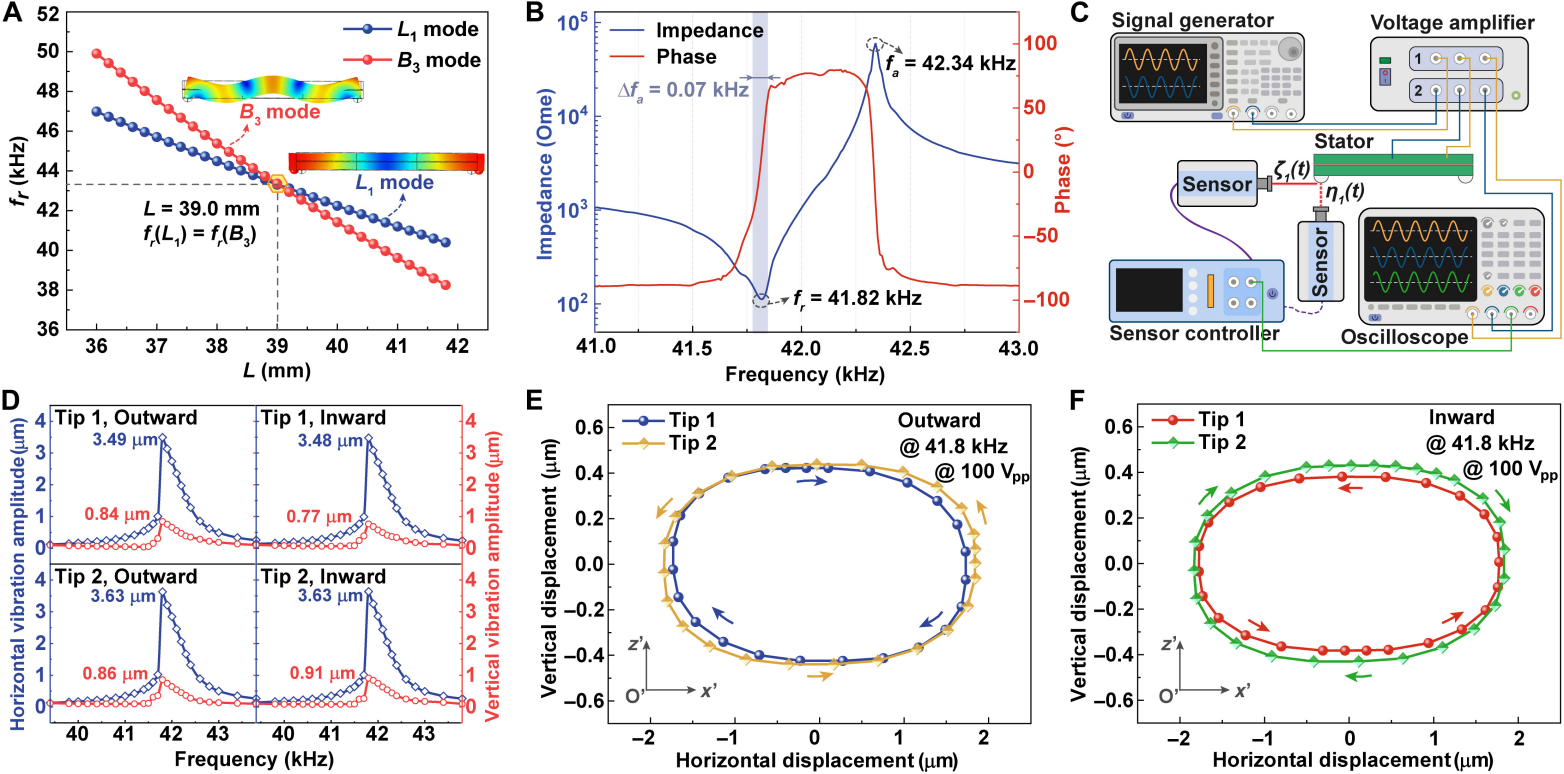
Performance verification of the *L*_1_–*B*_3_ stator. (A) The simulated resonant frequencies of *L*_1_ and *B*_3_ modes as a function of the length *L*. (B) Impedance magnitude and phase spectra. (C) Schematic diagram of test system when measuring the horizontal and vertical displacement responses. (D) Frequency dependence of the measured horizontal and vertical vibration peak–peak amplitudes of two friction tips for both outward and inward movements under the voltage of 100 V_pp_. Motion trajectories of two friction tips in the outward direction (E) and inward direction (F) at the frequency of 41.8 kHz.

When *W*, *T_p_*, and *T_c_* are fixed at 5 mm, 2 mm, and 0.4 mm, respectively, the resonant frequencies of *L*_1_ and *B*_3_ modes (*f_r,L1_* and *f_r,B3_*) under different *L* are illustrated in Fig. 2A. It is clear that *f_r,L1_* matches *f_r,B3_* well when *L* = 39.0 mm, and the corresponding frequency was calculated to be 43.4 kHz. Thus, the detailed dimensions of the piezoelectric stator can be determined, as indicated in Fig. S2.

Stress distribution is also an important index to evaluate the stability of the *L*_1_–*B*_3_ SLPUM. By means of FEM, as long as the voltage is lower than 350 V_pp_, the maximum von Mises stress of *L*_1_ and *B*_3_ modes are calculated to be smaller than the allowable stress of PZT-4 and copper when the factor of safety is set as 2 [38,39], verifying its long-term working ability (see Figs. S3 and S4). Therefore, the prototype specimen can be prepared on the basis of the simulation results.

The impedance magnitude and phase spectra of the piezoelectric bar vibrator around resonant states when exciting *L*_1_ and *B*_3_ modes simultaneously were firstly experimentally measured using an impedance analyzer (HP 4294A, Agilent), as shown in Fig. 2B. The detailed experimental procedure and results to verify the frequency degeneration are illustrated in Text S3 and Fig. S5. The frequency degeneration, i.e., the coupled mode between *L*_1_ and *B*_3_ modes was realized successfully as only one peak appears at 41.82 kHz, which is in good agreement with the numerical result and demonstrates the feasibility and accuracy of the FEM. To evaluate the energy conversion level from electrical energy to mechanical energy of the *L*_1_–*B*_3_ stator, the effective electromechanical coupling factor *k_eff_* and mechanical quality factor *Q_m_* can be calculated by [40]:

$$k_{\mathrm{eff}}=\sqrt{\frac{f_{a}^{2}-f_{r}^{2}}{f_{a}^{2}}}$$ (12)

$$Q_{m}=\frac{f_{r}}{∆f}$$ (13)

where *f_r_* and *f_a_* are resonant and anti-resonant frequencies, and ∆*f* denotes the half-power bandwidth. According to the tested impedance results, *k_eff_* is computed to be a reasonable value of about 16%, and *Q_m_* is as high as 597, which is almost the same as the mechanical quality factor of the PZT-4 ceramic (~600).

Then, a laser Doppler vibrometer (LV-S01, Sunny) with a displacement resolution of 15 pm and a sample rate of 15 × 10^6^ samples per second was employed to quantitatively evaluate the horizontal displacements along the *x*-axis and vertical displacements along the *z*-axis of the two friction tips under varying frequencies. During measurements, dual-channel AC drive signals with the voltage peak–peak amplitude of 100 V_pp_ were generated by a function generator (AFG3200B, Tektronix), amplified using a high-voltage amplifier (2350, Tegam), and monitored by a mixed signal oscilloscope (MSOX4024A, Keysight) (see Fig. 2C). Moreover, the phase difference between two AC signals applied on CH1 and CH2 was set as 90° and −90° for outward and inward movements, respectively. As illustrated in Fig. 2D, the maximum horizontal and vertical vibration amplitudes of both friction tips in both directions are all obtained at the frequency of 41.8 kHz.

Extracting the vibration responses at 41.8 kHz, the elliptical motion trajectories of these two friction tips within a period are depicted in Fig. 2E and F, with the directions represented by the arrows. We can see that for a specific motion (i.e., outward movement or inward movement), the two friction tips vibrate in opposite directions horizontally while synchronous vertically, which verifies the operating principle of the proposed stator operating in *L*_1_–*B*_3_ mode. In addition, the detailed displacement amplitudes of the motion trajectories of two friction tips for both outward and inward movements are listed in Table S2, with the amplitudes along horizontal and vertical directions measured to be about 3.5 μm and 0.85 μm, respectively, demonstrating the promise for high-speed movement. It can be calculated that for the outward movement, the differences of horizontal and vertical displacement amplitudes between the friction tip 1 and the friction tip 2 are 6% and 2.3%, respectively, and for inward movement, the corresponding differences are 3% and 11.6%. The differences are rational because of the unavoidable assembly or fabrication errors, such as the contact interface bonding between the friction tips and the piezoceramic plate, the clamping condition of the piezoelectric stator, etc. At the same time, trajectory amplitudes of the friction tip 1 vary slightly when changing its movement direction. As shown in Fig. 2E and F, the variations of 2.5% in the horizontal amplitude and 10.6% in the vertical amplitude were observed. Again, the non-full overlapping bidirectional motion trajectories should be attributed to the slight error of tip assembly.

### Actuation experiments

**Fig. 3.**
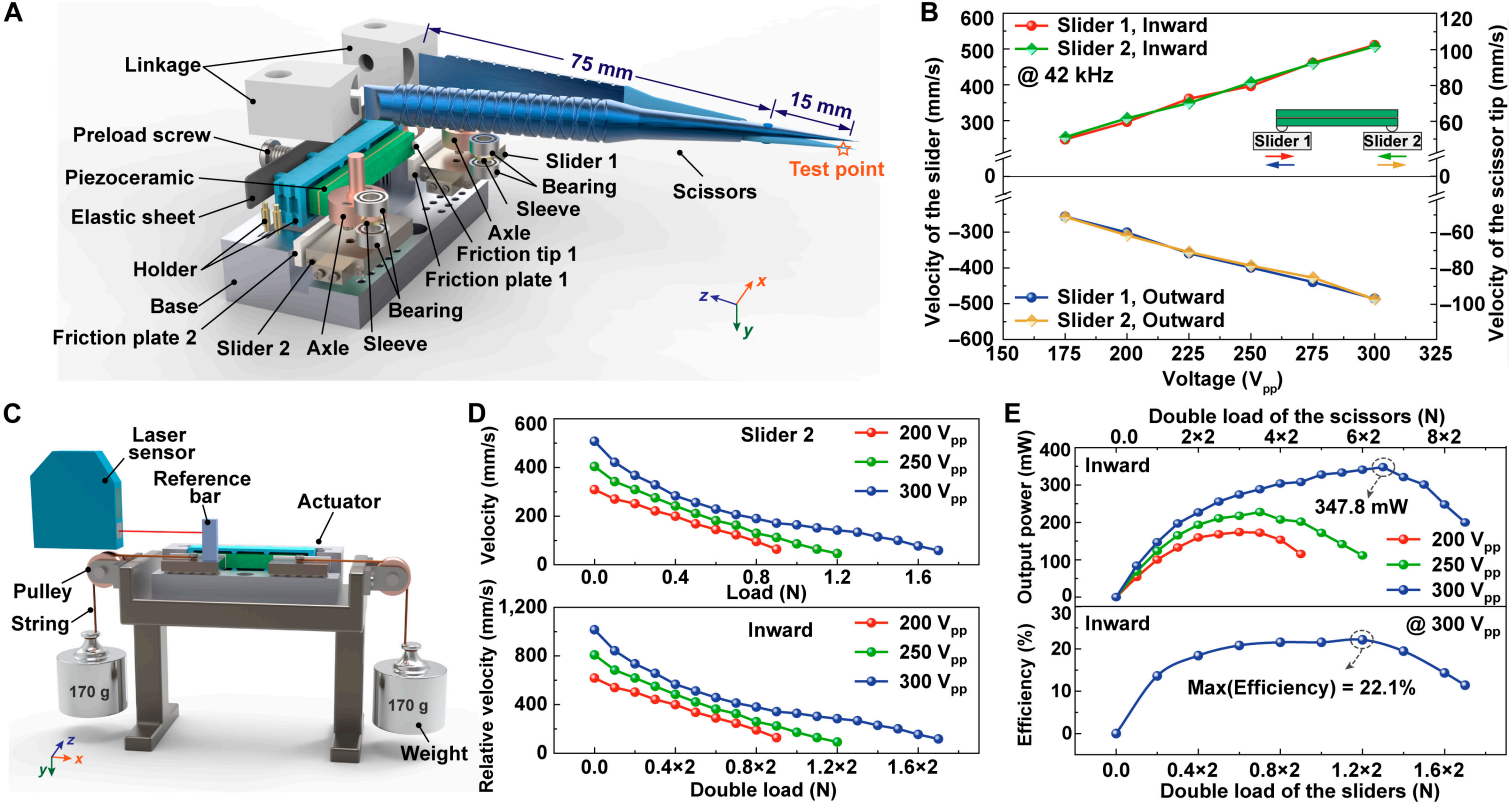
Output performances of the *L*_1_–*B*_3_ SLPUM working in the continuous-motion mode. (A) The exploded view of the proposed SLPUM prototype. (B) No-load velocity of the sliders as a function of driving voltage in outward and inward directions at 42 kHz. (C) The graphical setup for measuring the load performances. (D) Plots of the driving velocity versus mechanical load under varying driving voltages. (E) The output power and the efficiency as a function of double load.

The above theoretical derivations, numerical simulations, and experimental tests have illustrated that the presented design can successfully generate symmetrical motion or actuation outputs in a monolithic piezoelectric stator, while its output performances as a linear piezoceramic motor need to be evaluated for practical applications. As shown in Fig. 3A and Fig. S6, a prototype of the proposed SLPUM consisting of the (2 × 3) arrayed piezoceramic bar stator operating in *L*_1_–*B*_3_ mode, a pair of commercial microsurgical scissors, two sliders attached with two zirconia friction plates, a holder, a preload part, and a base were fabricated to confirm the symmetrical driving performances. The materials of each component are listed in Table S3. The piezoceramic bar-type stator is elastically pressed against one pair of standardized sliders (BWU 12-20, IKO) via its two friction tips by the preload part. Symmetric moving or actuating outputs outward or inward can be generated directly without using any complex transmission mechanisms. According to this structure, the linear strokes of two sliders and two scissor blade tips are 39.0 mm and 7.8 mm, respectively. As a comparison, previous reports have revealed that a multilayer piezoceramic actuator can be used for driving two arms moving symmetrically via a complex displacement amplification mechanism in a tweezer; the generated relative displacements are very limited (<100 μm) [41,42]. Sticking a reflective paper on the moving parts, their relative moving velocities can be tested by using the experimental system as shown in Fig. S7. An infrared thermometer (AS8204, Smart Sensor) was also used to monitor the piezoceramic bar’s temperature stability during the whole measurement processes.

Firstly, the influences of working frequency, applied voltage amplitude, and mechanical load on the moving velocity of the two sliders and scissor blade tips in the continuous-motion mode were analyzed systematically to determine the optimal working status. The preload screw was used to adjust the preload force applied to the *L*_1_–*B*_3_ stator, and the relationships between output performances (i.e., no-load velocity and double load of the sliders) and preload force are illustrated in Fig. S8. With the increase of the preload force, the no-load velocity and load capacity both tend to rise and then fall, and reach the maximum values at the preload force of 22 N and 25 N, respectively. To get better load capacity, a preload force of 25 N was chosen in this work.

As disclosed in Fig. S9, one pair of microsurgical scissors can be driven in both outward and inward directions stably within the frequency range of 41.6 kHz to 42.9 kHz under the voltage of 200 V_pp_. The velocity of inward movement is set as a positive value, while the velocity of outward movement is set as a negative value. It is clear that the motion velocities in two directions reach the maximum values at the frequency of 42 kHz, which can be chosen as the optimal resonant driving frequency *f_r_*. It should be noted that since the lever ratio of the scissors around their rotational axis is 1:5, there is always a 1:5 relationship between the velocity of the selected test point at the end of the scissor blade tips and that of the sliders.

At this frequency *f_r_*, the relationships between velocities and driving voltages in 2 directions are plotted in Fig. 3B. Obviously, the driving force provided by the piezoceramic bar stator is greater than the preload-induced static friction force when the applied voltage exceeds 175 V_pp_, so that the velocity of sliders rises nearly linearly with the increase of voltage, indicating its good controllability and repeatability in two moving directions. When the input voltage amplitude is 300 V_pp_, the velocities of each slider can reach more than 500 mm/s, which means that the relative motion velocity between the two sliders is as high as 1 m/s. This high-speed operation capability is closely related to the large horizontal vibration amplitudes of friction tips, and will definitely expand the application scenarios of the SLPUM. In addition, the four sets of velocity curves almost overlap, confirming the consistency of the vibration trajectories of two friction tips in two directions.

Then, the string–pulley–weight mechanism was employed to test the load capacity of the *L*_1_–*B*_3_ SLPUM under various voltages at 42 kHz. As depicted in Fig. 3C, weights of the same mass were suspended on two sliders by strings and pulleys, and a reference bar was bonded on slider 2 to assist in measuring the velocity in the inward direction. Figure 3D shows the negative correlation between the velocities of slider 2 and the pulling loads. When the signal voltage is 300 V_pp_, the maximum mechanical load of slider 2 was tested to be 1.7 N, while the inward pulling load or inward driving force of two sliders of the *L*_1_–*B*_3_ SLPUM was tested to be 3.4 N (2×1.7 N). Therefore, the mechanical load of the pair of scissors can be calculated to be as high as 17 N (2 × 8.5 N), as long as the material is strong enough. The maximum total output power of the SLPUM is 347.8 mW (see Fig. 3E), and the corresponding power density is as high as 405.4 mW/cm^3^ or 9.65 mW/cm^3^·kHz, which is more than double that of the typical LPUMs. This high power density further demonstrates the competitive advantages of the proposed brand-new SLPUM operating in *L*_1_–*B*_3_ mode and the symmetrical driving mechanism that fully exploits the overall capacity of the piezoceramic bar stator.

Figure 3E also shows the measured energy conversion efficiency of the SLPUM as a function of mechanical load under the voltage of 300 V_pp_, and the input power consumption is plotted in Fig. S10. It is found that the efficiency of the SLPUM reaches the maximum value of about 22.1% when the dual-output load of the sliders (or the scissors) is 2.4 N (or 12 N). Apparently, the high efficiency is attributed to the large *Q_m_*, low temperature rise, and the advantageous symmetrical actuating mechanism.

**Fig. 4.**
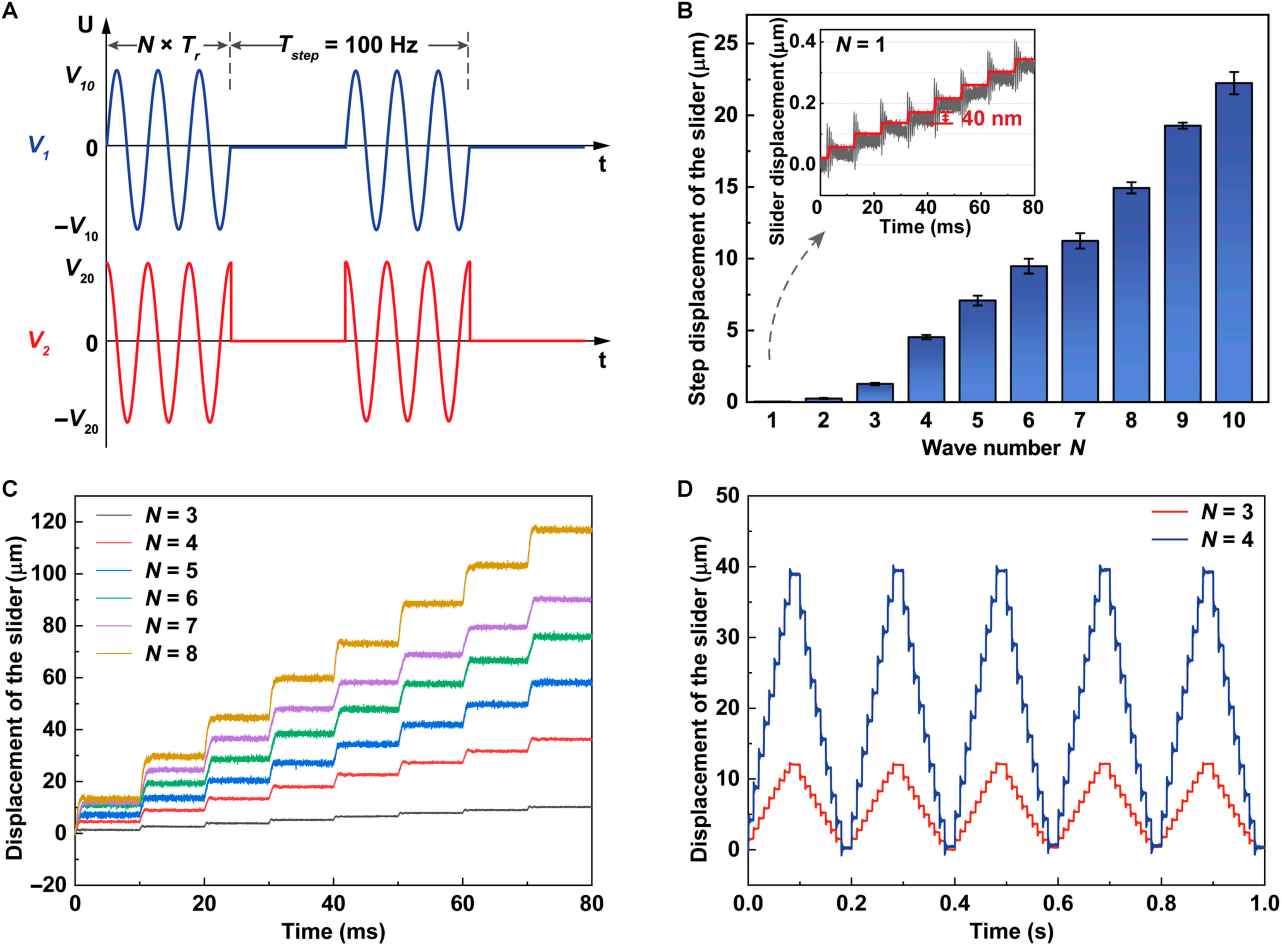
Stepping characteristics of one slider of the *L*_1_–*B*_3_ SLPUM when *f_r_* = 42 kHz and *f_step_* = 100 Hz. (A) The driving voltage signals of the stepping mode. (B) The plot of the step displacements versus *N*, and illustration of the step motions of the slider with the resolution of 40 nm when *N* = 1. (C) The real-time displacement responses with varying *N*. (D) The local step servo motions with no drift phenomenon.

The stepping resolution is also a momentous performance indicator of the motor. As depicted in Fig. 4A, the resonance frequency *f_r_* (= 1/*T_r_*), voltage peak–peak amplitude 2*V*_10_ or 2*V*_20_, and step motion frequency *f_step_* (= 1/*T_step_*) of two intermittent sinusoidal excitation signals with a phase difference of 90° were set as 42 kHz, 300 V_pp_, and 100 Hz, respectively. By controlling the wave number *N* per step, not only can the duty factor (= *N*×*T_r_*/*T_step_*) be tuned, but also different step displacements can be generated, as shown in Fig. 4B. To reveal the controllability and repeatability of the step motions in every step cycle, the measured real-time displacement responses of the sliders under varying *N* in open-loop control are also illustrated in Fig. 4C. As expected, the *L*_1_–*B*_3_ SLPUM can achieve higher stepping resolution when decreasing *N*, and the minimum step displacement amplitude of the slider was observed to be as low as 40 nm (see Fig. 4B), indicating that the motor accumulated enough energy to overcome the friction force to generate step motions in a very short time when *N* = 1. Theoretically, the stepping resolution of the scissor blade tips can reach a minimum of 8 nm according to the lever ratio. It is clear that although there is obvious environment noise as the experiments were carried out without a precise vibration isolation table, the red motion trajectory of one slider extracted from the raw response data is still stable and repeatable. There is a subtle difference between the step displacements, but it is rational because the step displacements are sensitive to the slight change at frictional coupling interfaces when operating in the open-loop condition. Moreover, it can be concluded that the relative moving velocity of the two direct outputs can be regulated in a quite wide range by adjusting the parameters of the exciting signals.

By applying two signals consisting of voltage cycles to CH1 and CH2, the local step servo motions under different *N* can be generated as shown in Fig. 4D. In this work, one voltage cycle lasting 0.2 s includes 10 step-out (for outward movement) and 10 step-in (for inward movement) voltage sequences. It can be clearly observed that the bidirectional local microscale-step servo motions are very stable; i.e., there is no drift phenomenon. Although the unevenness of stepping motions increases during starting and stopping, this is inevitably caused by the acceleration and deceleration of the SLPUM when it changes the driving direction, and it can be ameliorated with the aid of the closed-loop control method in the future.

**Fig. 5.**
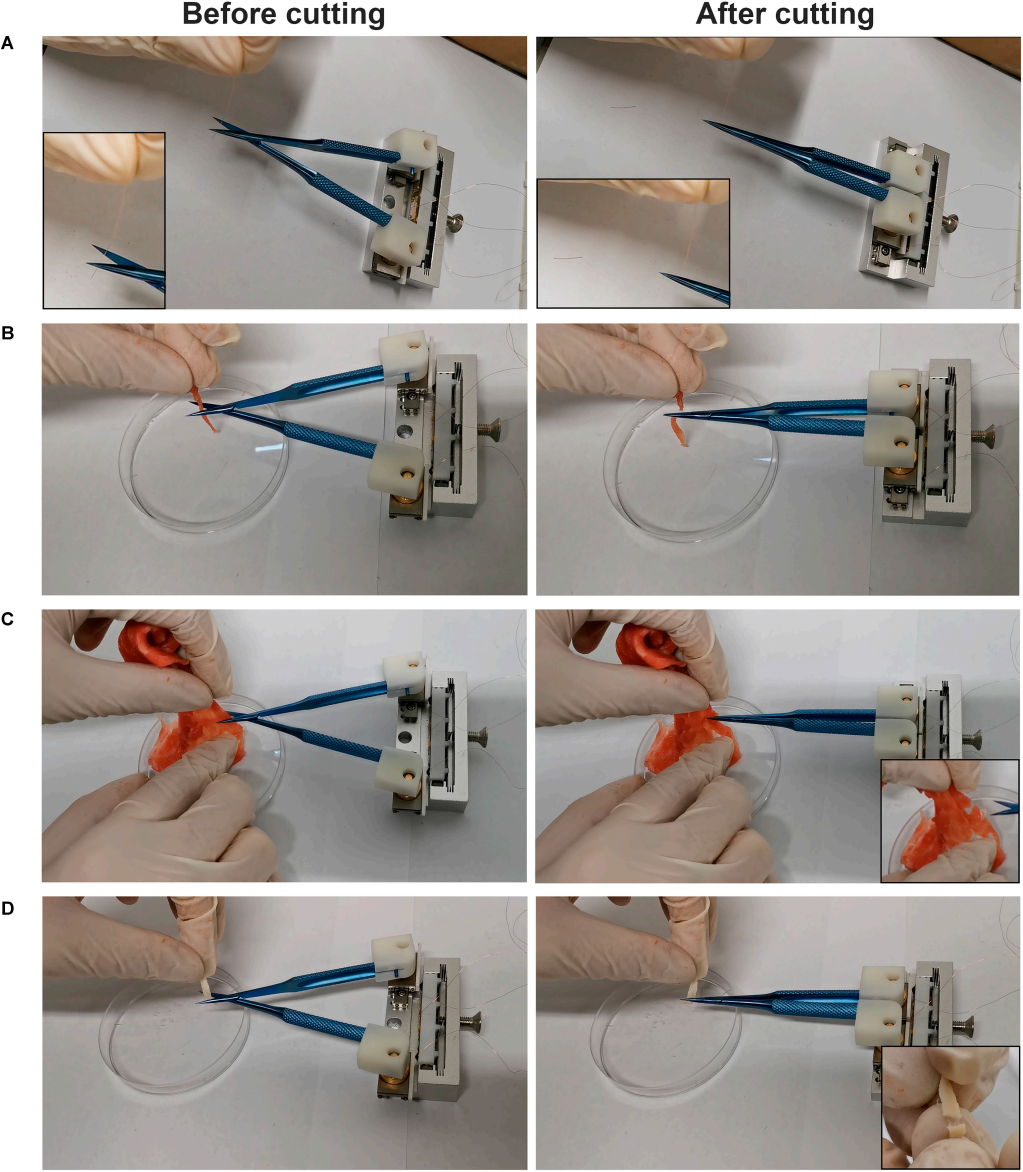
Photos of the pair of microsurgical scissors cutting copper wires (A), pork (B), beef slices (C), and intestines (D).

As one pair of commercial microsurgical scissors was mounted on the sliders, the *L*_1_–*B*_3_ SLPUM could be further applied to microsurgical robots for performing high-precision microsurgical operations, such as cataract microsurgery or cerebral thrombosis microsurgery. For verification, we used this prototype to conduct experiments in different application scenarios, such as cutting copper wires, pork, beef slices, intestines, etc., as shown in Fig. 5 and Movie S1. By changing the frequency, amplitude, step motion frequency, and wave number of the voltage signals, the approaching speed, resolution, and tip shearing force of the two scissor blades can be controlled. If the end-effector is replaced by a pair of medical tweezers, clamps, or other tools, further micromanipulation can be accomplished.

It should be noted that the inherent hysteresis and creep phenomena originating from the ferroelectricity of PZT-4 material have no influence on the positioning task though the proposed actuator operates in the open-loop condition, because it converts the high-frequency micro-amplitude vibration of the piezoceramic bar stator into linear movements of two sliders and maintains the position through self-locking performance with no input signals after reaching the designated position. Undoubtedly, the implementation of high positioning accuracy and steady stepping motion will make the *L*_1_–*B*_3_ SLPUM more competitive in the micro/nano operation actuating technology.

The optimal operating conditions and optimum mechanical output performances of the *L*_1_–*B*_3_ SLPUM are compared with several previously reported piezoelectric actuators, including the bolt-clamped metal–ceramic, the bulk-type ceramic or bonded-type metal–ceramic, and co-fired ceramic types, as summarized in Table. Obviously, in addition to the direct symmetrical actuation function and double outputs of two-way motions using only one piezoceramic bar, the proposed motor exhibits excellent comprehensive actuation properties, such as compact structure, fast velocity, high positioning resolution, large power density, wide controllable velocity range, and high efficiency. Moreover, this actuator shows a long working life without apparent temperature rise (no more than 20 °C), and there is no apparent performance degradation as long as the input electric field is within the safe level. In fact, according to reported technique parameters, the service lifetime of PUMs could be over 3,000 h [43]. Considering the advantages of the closed-loop control method, it can be reasonably inferred that higher stepping resolution and greater repeatability can be obtained if the closed-loop control is adopted. In future work, we also plan to integrate a position sensor for realizing closed-loop control of the SLPUM.

**Table.** Main performance comparison between the proposed *L*_1_–*B*_3_ SLPUM and previously reported LPUMS.

Parameters	Ref*.* [27]	Ref. [33]	Ref. [44]	Ref*.* [45]	Ref*.* [46]	This work
Function of two-way symmetrical motions?	No	No	No	No	No	Yes
Structure type	Bulk ceramic	Co-fired ceramic	Bulk ceramic	Bolt-clamped metal–ceramic	Bonded metal–ceramic	Bonded metal–ceramic
Voltage (V_pp_/mm)	130	400	75	75	282	150
Working frequency (kHz)	59.1	125.1	92	22.35	54.2	42.0
Volume of driving elements (mm^3^)	900	231	450	~180,830	~6,615	858
Maximum moving velocity (mm/s)	211.2	123.7	230	572	765	511 + 507 (sliders)
						102.2 + 101.4 (scissors)
Maximum output power (mW)	218.3	53.5	60	2,513	1,200	347.8
Power density (mW/cm^3^)	242.6	231.6	133.3	13.9	181.4	405.4
Power density (mW/cm^3^·kHz)	4.1	1.85	1.45	0.62	3.35	9.65
Stepping resolution (nm)	33	50	Not provided	2,000	Not provided	40 (slider)
Efficiency	10.6%	Not provided	12%	Not provided	Not provided	22.1%

## Discussion

In conclusion, we report a brand-new SLPUM equipped with a pair of microsurgical scissors based on an (2 × 3) arrayed, compact piezoelectric ceramic bar stator operating in *L*_1_–*B*_3_ mode. The elaborate *L*_1_–*B*_3_ SLPUM can generate two symmetrical and synchronous outward/inward linear motion outputs at the same velocity via one pair of sliders without using an additional mechanical transmission mechanism. The prototype shows the following features: (a) fast relative moving velocity (~1.0 m/s) of two sliders in outward or inward direction, (b) high step resolution (40 nm for the slider), (c) relatively large output force (3.4 N for the sliders and 17 N for the scissors), (d) high output power (347.8 mW) and power density (405.4 mW/cm^3^ or 9.65 mW/cm^3^·kHz), and (e) high efficiency (22.1%) under the electric field of 150 V_pp_/mm.

This work also demonstrates that a linear piezoelectric ultrasonic motor operating in symmetrical driving principle is more rational and twice as efficient in comparison with conventional piezoelectric ultrasonic motors, but it has not been revealed before. Therefore, this work is instructive for future piezoelectric actuating device designs.

## Methods

### Finite element simulation

All FEM simulations of the *L*_1_–*B*_3_ stator were carried out by using the module of piezoelectric device in COMSOL Multiphysics. The three-dimensional geometrical models of the stator were first built according to the design requirements. The ceramic material PZT-4 was the default material in the built-in material libraries, while its *d*_33_, *d*_31_, and *d*_15_ coefficients were altered to 280, −100, and 420 pm/V according to the commercialized PZT-4 we used. Each piezo-plate was divided into three parts, and the polarization directions of each part were set as shown in Fig. 1A.

### Parameter measurement

The impedance magnitude and phase spectra of the *L*_1_–*B*_3_ stator were measured by a precise impedance analyzer (HP 4294A, Agilent). The dual-channel sinusoid-wave signals were generated by a function generator (AFG3200B, Tektronix), amplified using a high-voltage amplifier (2350, Tegam), and monitored by a mixed signal oscilloscope (MSOX4024A, Keysight). Then, the actuation characteristics were measured using the high-precision laser Doppler vibrometer (LV-S01, Sunny).

## Data Availability

All data needed to evaluate the conclusions in the paper are present in the paper and/or the Supplementary Materials. Additional data related to this paper may be requested from the authors.

## Supplementary Materials

Supplementary 1 Text S1. The expressions of each coefficient of the longitudinal and lateral vibration displacements. Text S2. Determination of the clamping positions on the piezoelectric stator. Fig. S1. The influence of different clamping positions on the actuating performances. Fig. S2. The detailed dimensions of the piezoelectric stator. Fig. S3. The maximum von Mises stress variation of both *L*_1_ and *B*_3_ modes with the applied voltages. Fig. S4. Distributions of von Mises stress under the voltage of 200 V_pp_. Text S3. Experimental verification of the resonant frequency degeneration. Fig. S5. Experimental verification of the frequency degeneration. Fig. S6. The assembly diagram of the proposed SLPUM prototype. Fig. S7. Photograph of the experimental setup composed of a drive module, a measurement module, and a monitoring module. Fig. S8. The relationships between the output performances (i.e., no-load velocity of slider 1 and double load of the sliders) and the preload force. Fig. S9. The relationship between the velocity and working frequency under the voltage of 200 V_pp_. Fig. S10. The input power consumption as a function of double load. Table S1. Main physical parameters of PZT-4 ceramic. Table S2. Displacement amplitudes of the motion trajectories of two friction tips for both outward and inward movements. Table S3. The materials of the motor components. Movie S1. Scissoring experiments of the *L*_1_–*B*_3_ SLPUM.
