# Supplementary material for: A Symmetric-Actuating Linear Piezoceramic Ultrasonic Motor Capable of Producing a Scissoring Effect
Source: Research (Wash D C). 2023 Jun 6;6:0156. doi: 10.34133/research.0156 (PMC10243895; doi:10.34133/research.0156)
Supplement: Supplementary 1 — Text S1. The expressions of each coefficient of the longitudinal and lateral vibration displacements. Text S2. Determination of the clamping positions on the piezoelectric stator. Fig. S1. The influence of different clamping positions on the actuating performances. Fig. S2. The detailed dimensions of the piezoelectric stator. Fig. S3. The maximum von Mises stress variation of both L1 and B3 modes with the applied voltages. Fig. S4. Distributions of von Mises stress under the voltage of 200 Vpp. Text S3. Experimental verification of the resonant frequency degeneration. Fig. S5. Experimental verification of the frequency degeneration. Fig. S6. The assembly diagram of the proposed SLPUM prototype. Fig. S7. Photograph of the experimental setup composed of a drive module, a measurement module, and a monitoring module. Fig. S8. The relationships between the output performances (i.e., no-load velocity of slider 1 and double load of the sliders) and the preload force. Fig. S9. The relationship between the velocity and working frequency under the voltage of 200 Vpp. Fig. S10. The input power consumption as a function of double load. Table S1. Main physical parameters of PZT-4 ceramic. Table S2. Displacement amplitudes of the motion trajectories of two friction tips for both outward and inward movements. Table S3. The materials of the motor components. Movie S1. Scissoring experiments of the L1–B3 SLPUM. [file research.0156.f1.zip › RevisedSupplementalMaterial_Highlighted_SLPUM_2nd.docx]

Supplementary Materials for

A Symmetric-Actuating Linear Piezoceramic Ultrasonic Motor Capable of Producing a Scissoring Effect

Zhanmiao Li^1^, Xingyu Yi^1^, Rongqi Zhu^1^, Zhonghui Yu^1^, Xiaoting Yuan^1^, MohammadJavad PourhosseiniAsl^1^, and Shuxiang Dong^1,2*^

^1^School of Materials Science and Engineering, Peking University, Beijing 100871, China.

^2^Institute for Advanced Study, Shenzhen University, Shenzhen 518051, China.

^*^Address correspondence to: sxdong@szu.edu.cn

This file includes:

Text S1. The expressions of each coefficient of the longitudinal and lateral vibration displacements.

Text S2. Determination of the clamping positions on the piezoelectric stator.

Fig. S1. The influence of different clamping positions on the actuating performances.

Fig. S2. The detailed dimensions of the piezoelectric stator.

Fig. S3. The maximum von Mises stress variation of both *L_1_* and *B_3_* modes with the applied voltages.

Fig. S4. Distributions of von Mises stress under the voltage of 200 V_pp_.

Text S3. Experimental verification of the resonant frequency degeneration.

Fig. S5. Experimental verification of the frequency degeneration.

Fig. S6. The assembly diagram of the proposed SLPUM prototype.

Fig. S7. Photograph of the experimental setup composed of a drive module, a measurement module, and a monitoring module.

Fig. S8. The relationships between the output performances (i.e., no-load velocity of the slider 1 and double load of the sliders) and the preload force.

Fig. S9. The relationship between the velocity and working frequency under the voltage of 200 V_pp_.

Fig. S10. The input power consumption as a function of double load.

Table S1. Main physical parameters of PZT-4 ceramic.

Table S2. Displacement amplitudes of the motion trajectories of two friction tips for both outward and inward movements

Table S3. The materials of the motor components.

Movie S1. Scissoring experiments of the *L_1_-B_3_* SLPUM.

Text S1. The expressions of each coefficient of the longitudinal and lateral vibration displacements.

For the Equation 6 and 7, the coefficients can be calculated as follows:

$\mu_{P}=\frac{2d_{31}}{s_{11}^{E}(\frac{2T_{p}}{s_{11}^{E}}+\frac{T_{c}}{s_{11c}})}$ (S1)

$\zeta_{0}\left( x \right)=\frac{\cos\left( kL-kx \right)-\cos kx}{k\sin kL}$ (S2)

$k=\omega\sqrt{\frac{\bar{\rho}}{\frac{n}{s_{11}^{E}}+\frac{1-n}{s_{11c}}}}$ (S3)

$\eta_{0}\left( x \right)=\frac{B\left( \sinh\beta x+\sin\beta x \right)+C\left( \cosh\beta x+\cos\beta x \right)+\cosh\beta x}{\Omega\beta^{2}}$ (S4)

$\varphi_{P}=\frac{ⅆ_{31}W(T_{p}+T_{c})}{s_{11}^{E}}$ (S5)

$B=\frac{\cosh\beta L\sin\beta L+\sinh\beta L\cos\beta L-\sinh\beta L-\sin\beta L}{2-2\cosh\beta L\cos\beta L}$ (S6)

$C=\frac{-\sinh\beta L\sin\beta L+\cosh\beta L\cos\beta L+\cosh\beta L-\cos\beta L-1}{2-2\cosh\beta L\cos\beta L}$ (S7)

$\Omega=\frac{T_{p}\left( 4T_{p}^{2}+6T_{p}T_{c}+3T_{c}^{2} \right)-3k_{31}^{2}T_{p}{(T_{p}+T_{c})}^{2}}{6s_{11}^{E}(1-k_{31}^{2})}W+\frac{T_{c}^{3}W}{12s_{11c}}$ (S8)

$\beta=\sqrt[4]{\frac{\omega^{2}\bar{\rho}(2T_{p}+T_{c})W}{\Omega}}$ (S9)

$\bar{\rho}=\frac{2\rho_{p}T_{p}+\rho_{c}T_{c}}{2T_{p}+T_{c}}$ (S10)

$n=\frac{2T_{p}}{2T_{p}+T_{c}}$ (S11)

where the coefficients $\mu_{P}$, $\varphi_{P}$, *k*, $\Omega$, and $\bar{\rho}$ have the physical significance of electromechanical conversion factor of the longitudinal vibration, electromechanical conversion factor of the lateral vibration, wavenumber, flexural rigidity, and average mass density of the three-layer laminate; *ρ_c_* and *T_c_* are the mass density and thickness of the copper sheet; and *ρ_p_*, *k_31_*, *L*, *W*, and *T_p_* represent the mass density, electromechanical coupling factor, length, width, and thickness of the piezoceramic plate.

Text S2. Determination of the clamping positions on the piezoelectric stator.

From the following Fig. S1, it can be seen that the middle two nodes of *B_3_* mode shape are more suitable for clamping the piezo-stator in order to reduce the influence on the vibrations of piezoceramic bar.


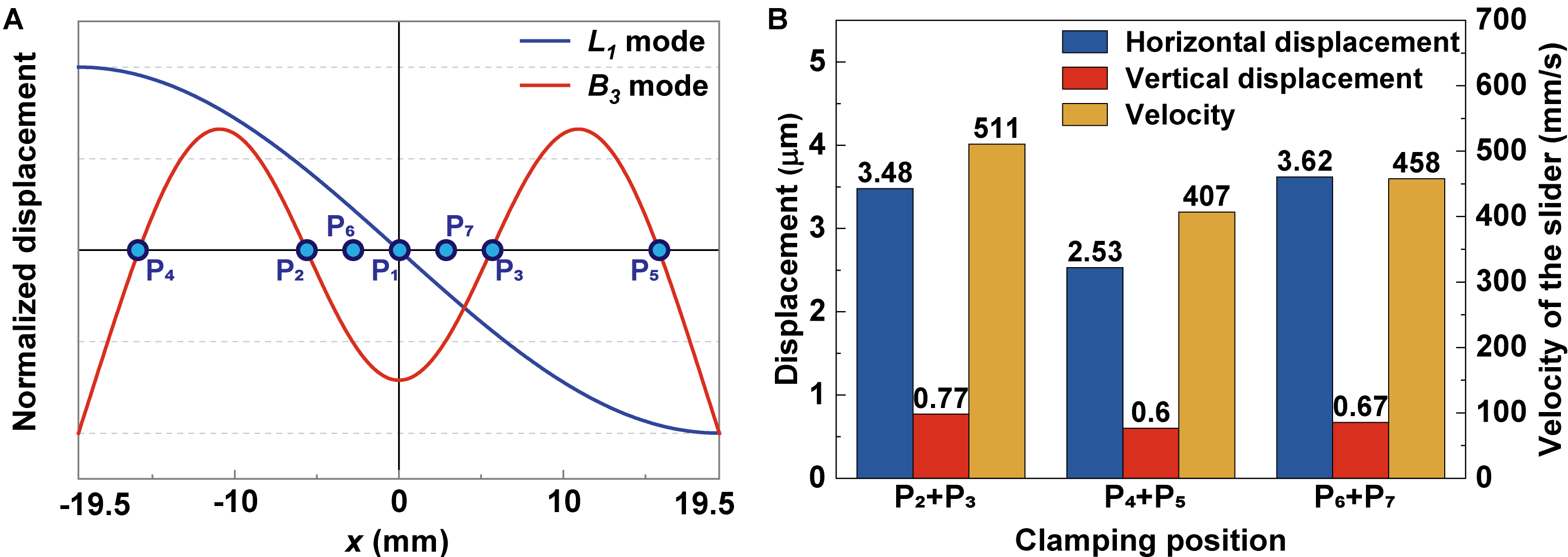


Fig. S1. The influence of different clamping positions on the actuating performances. (A) The chosen clamping positions P_1_ ~ P_7_. (B) The horizontal and vertical displacements of the friction tip and velocity of the slider 1 under the condition of different clamping positions.


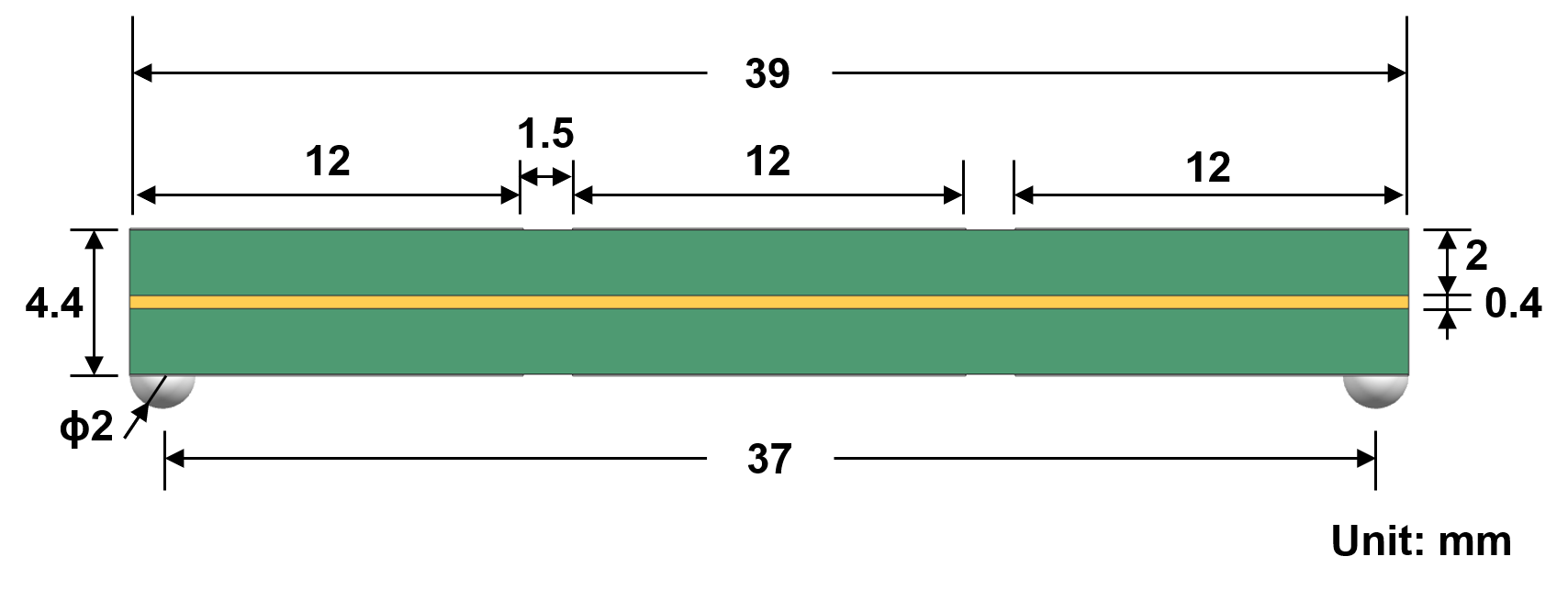


Fig. S2. The detailed dimensions of the piezoelectric stator.


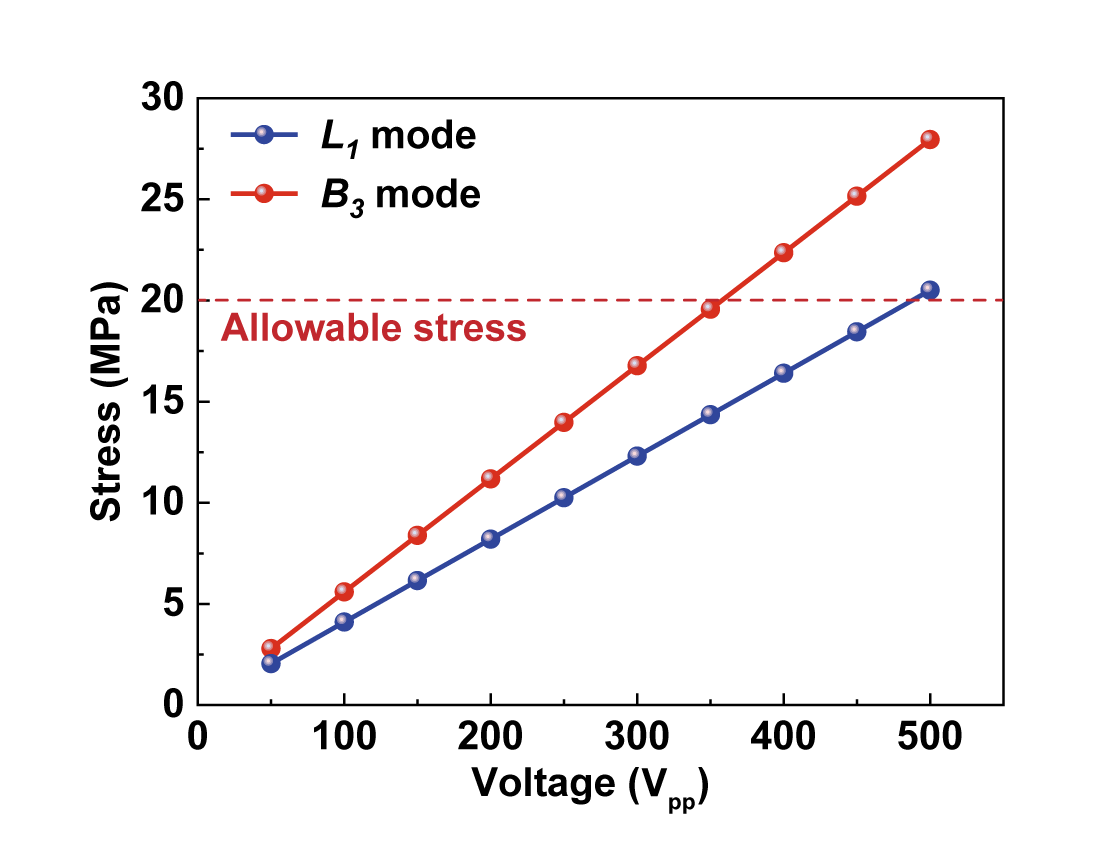


Fig. S3. The maximum von Mises stress variation of both *L_1_* and *B_3_* modes with the applied voltages.


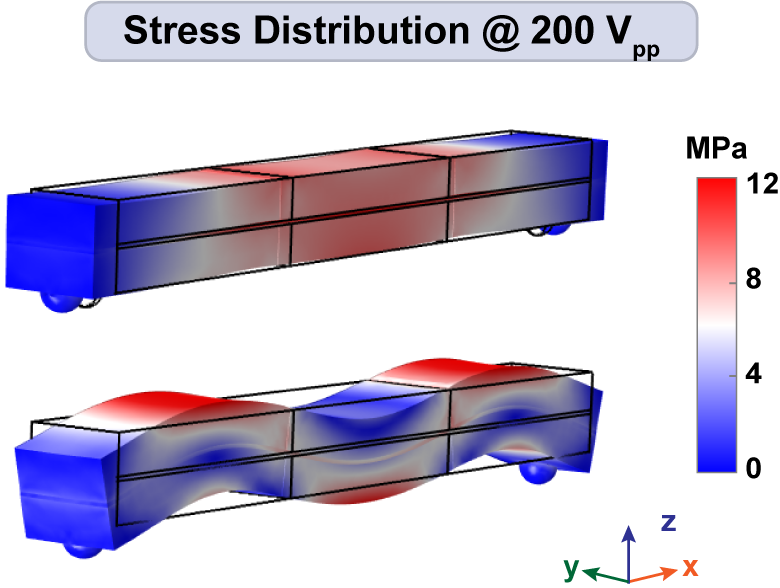


Fig. S4. Distributions of von Mises stress under the voltage of 200 V_pp_.

Text S3. Experimental verification of the resonant frequency degeneration.

As shown in Fig. S5, it is found that there is only a difference of 30 Hz between the resonant frequencies of *L_1_* mode and *B_3_* mode, so the frequency degeneration was realized successfully when they were excited at the same time and only one resonant peak appears at 41.82 kHz (see Fig. 2B).


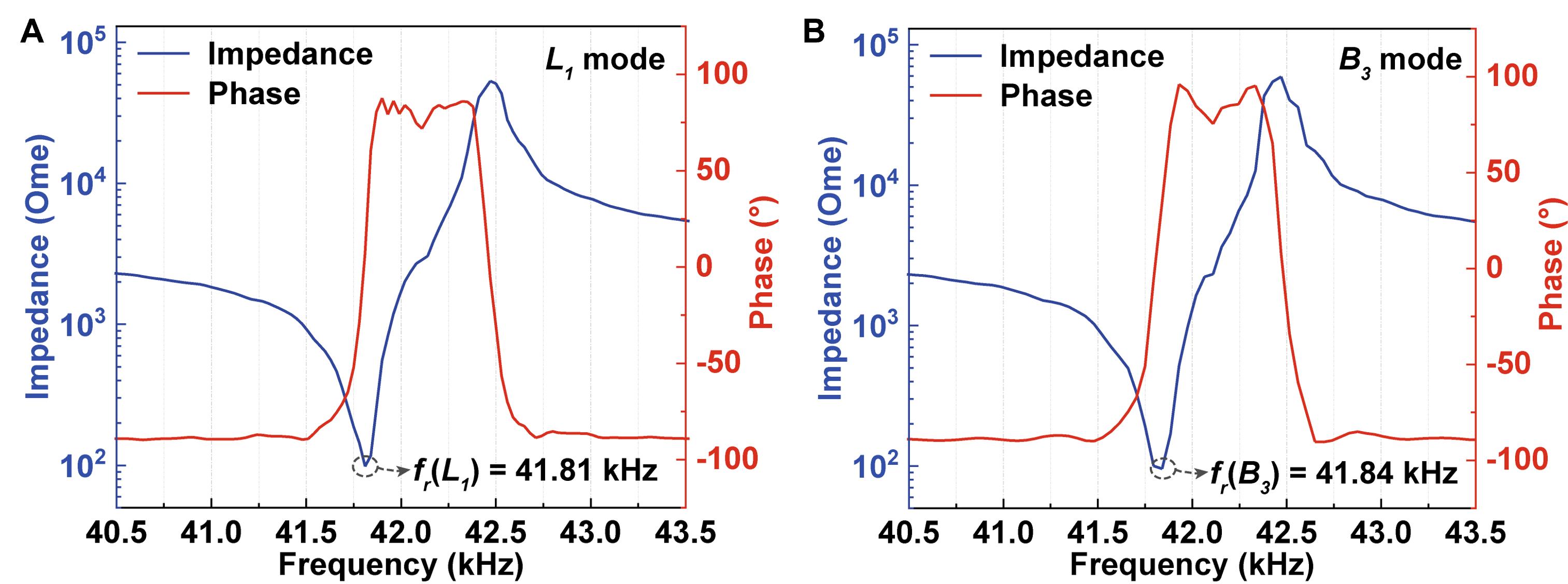


Fig. S5. Experimental verification of the frequency degeneration. Impedance magnitude and phase spectra of *L_1_* mode (A) and *B_3_* mode (B).


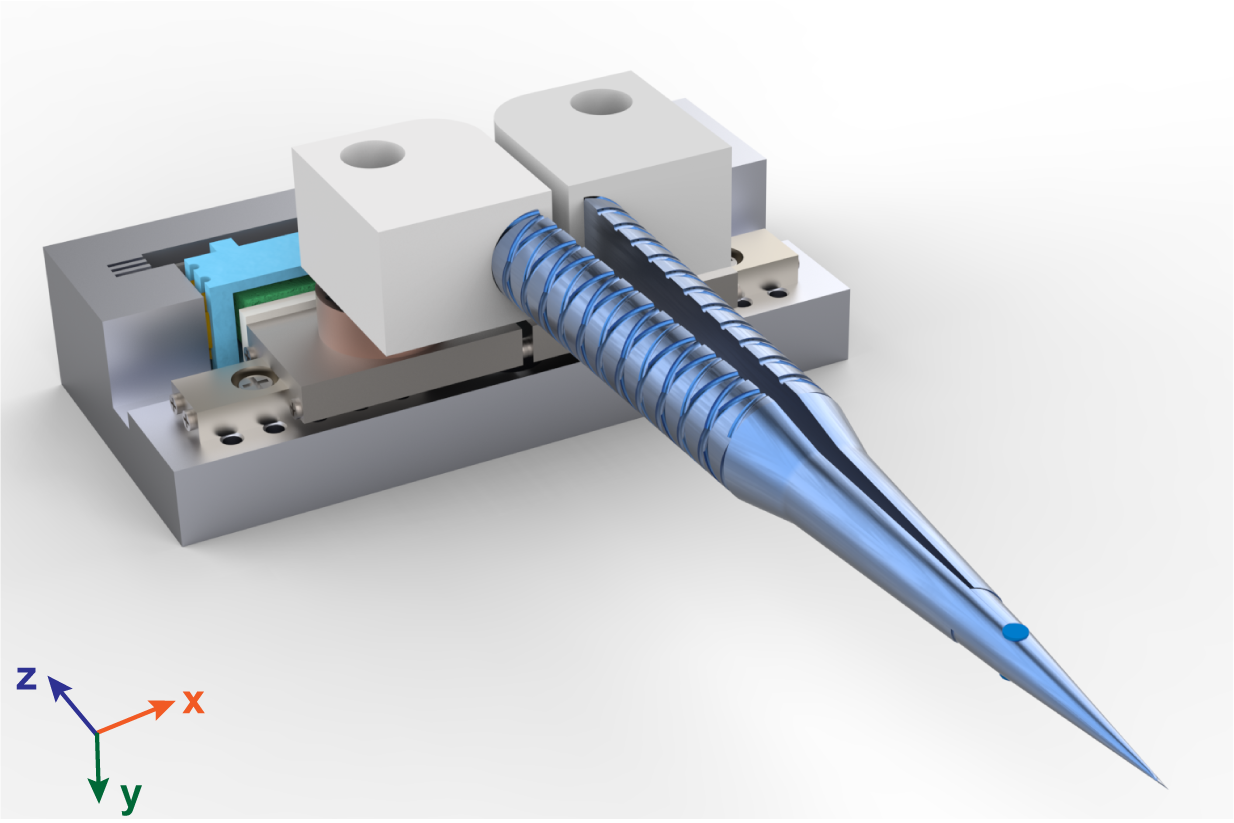


Fig. S6. The assembly diagram of the proposed SLPUM prototype.


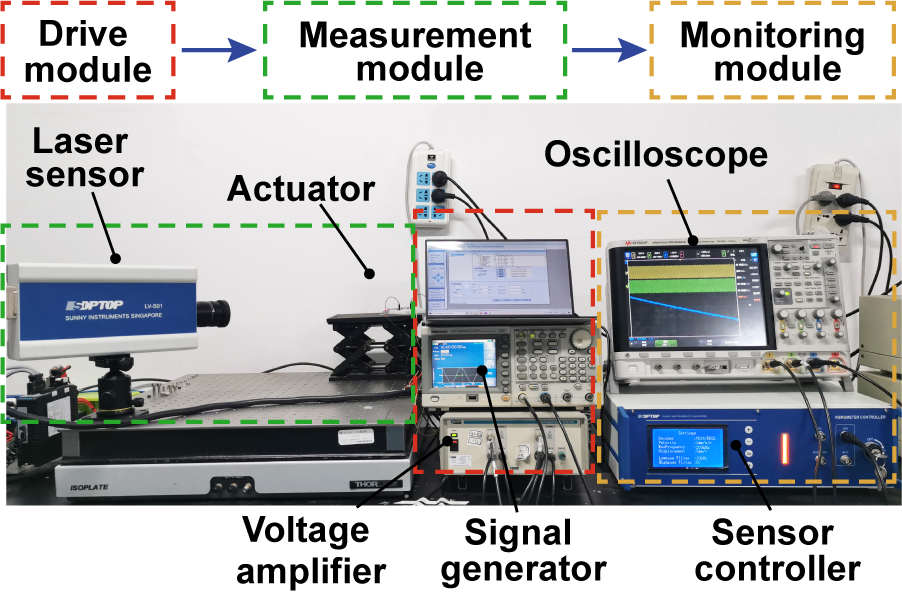


Fig. S7. Photograph of the experimental setup composed of a drive module, a measurement module, and a monitoring module.





Fig. S8. The relationships between the output performances (i.e., no-load velocity of the slider 1 and double load of the sliders) and the preload force.


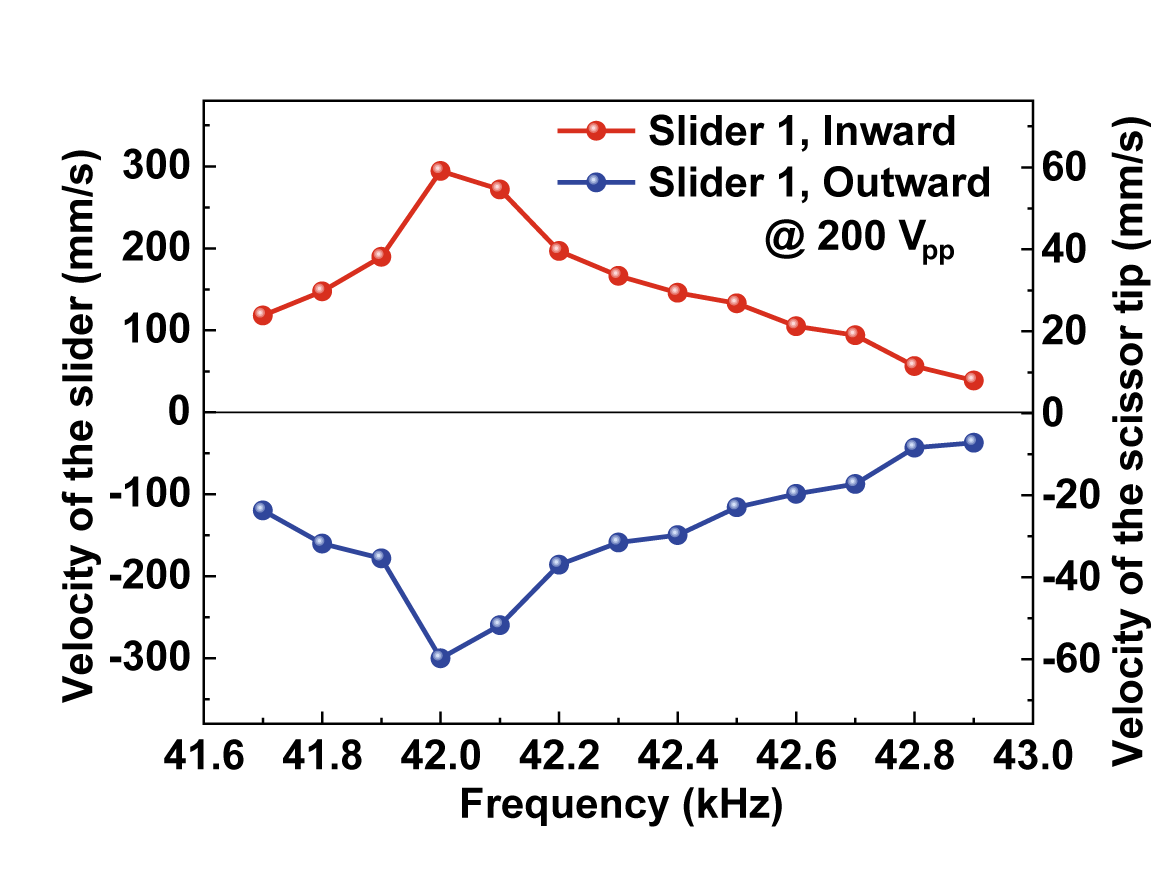


Fig. S9. The relationship between the velocity and working frequency under the voltage of 200 V_pp_.


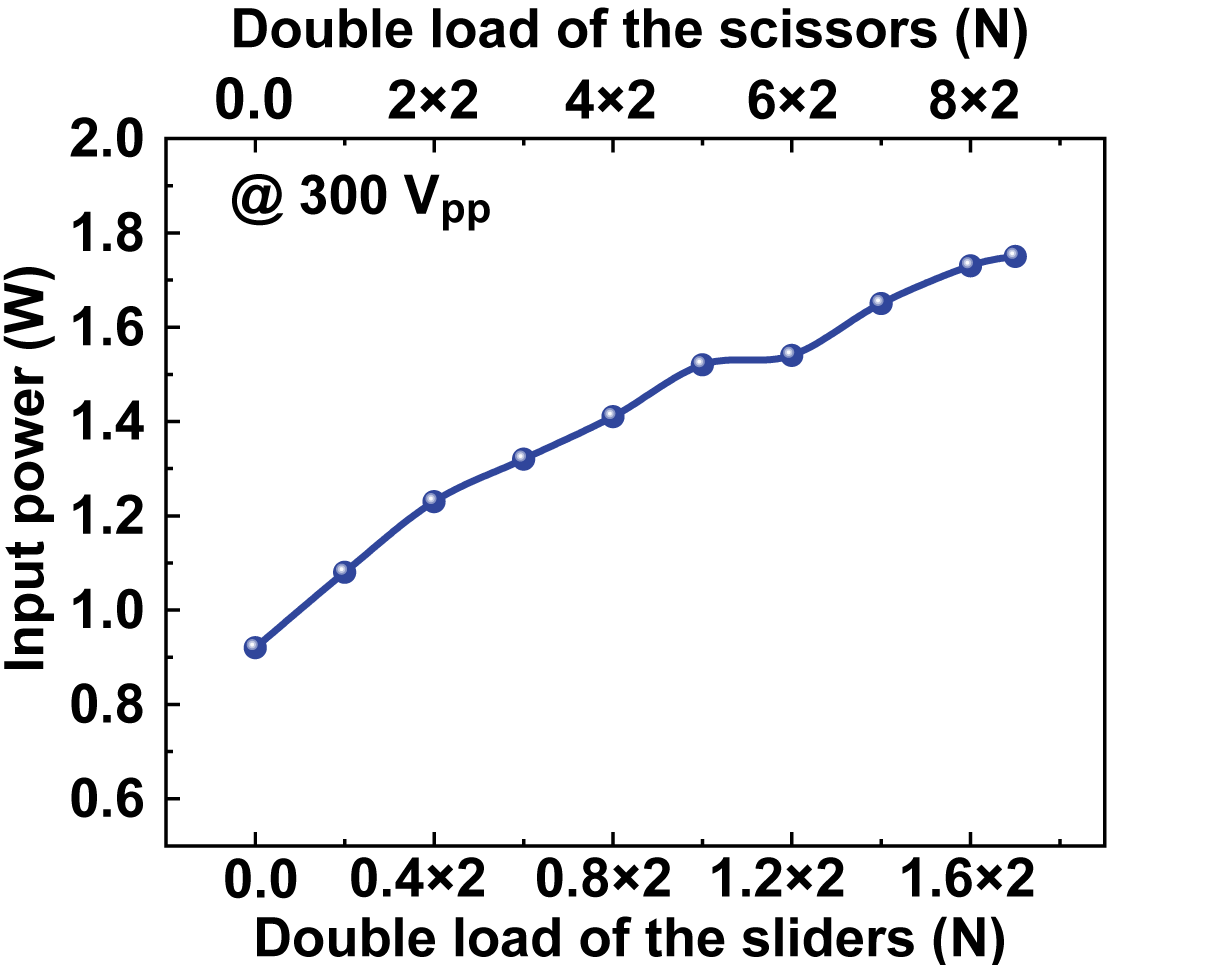


Fig. S10. The input power consumption as a function of double load.

Table S1. Main physical parameters of PZT-4 ceramic

| Physical parameters | Values |
| --- | --- |
| Piezoelectric matrix [*d*] | $\left[ \begin{matrix} 0 & 0 & \begin{matrix} 0 & 0 & \begin{matrix} 4.2 & 0 \end{matrix} \end{matrix} \\ 0 & 0 & \begin{matrix} 0 & 4.2 & \begin{matrix} 0 & 0 \end{matrix} \end{matrix} \\ -1.0 & -1.0 & \begin{matrix} 2.8 & 0 & \begin{matrix} 0 & 0 \end{matrix} \end{matrix} \end{matrix} \right]\times{10}^{-10} C/N$ |
| Stiffness matrix [*c^E^*] | $\left[ \begin{matrix} \begin{matrix} 13.9 & 7.8 & 7.4 \\ 7.8 & 13.9 & 7.4 \\ 7.4 & 7.4 & 11.5 \end{matrix} & \begin{matrix} 0 & 0 & 0 \\ 0 & 0 & 0 \\ 0 & 0 & 0 \end{matrix} \\ \begin{matrix} 0 & 0 & 0 \\ 0 & 0 & 0 \\ 0 & 0 & 0 \end{matrix} & \begin{matrix} 2.6 & 0 & 0 \\ 0 & 2.6 & 0 \\ 0 & 0 & 3.1 \end{matrix} \end{matrix} \right]\times{10}^{10} N/m^{2}$ |
| Relative dielectric matrix [$\varepsilon_{r}^{T}$] | $\left[ \begin{matrix} \begin{matrix} 1475 & 0 \\ 0 & 1475 \end{matrix} & \begin{matrix} 0 \\ 0 \end{matrix} \\ \begin{matrix} 0 & 0 \end{matrix} & 1200 \end{matrix} \right]$ |

Table S2. Displacement amplitudes of the motion trajectories of two friction tips for both outward and inward movements

|  | Outward direction | | Inward direction | |
| --- | --- | --- | --- | --- |
|  | Horizontal amplitude (μm) | Vertical amplitude (μm) | Horizontal amplitude (μm) | Vertical amplitude (μm) |
| Friction tip 1 | 3.46 | 0.85 | 3.55 | 0.76 |
| Friction tip 2 | 3.68 | 0.87 | 3.66 | 0.86 |

Table S3. The materials of the motor components

| Components | Material |
| --- | --- |
| Piezoceramics | PZT-4 |
| Friction plates | Zirconia |
| Friction tips | Zirconia |
| Holder | Ductile resin / Brass |
| Base | Aluminium alloy |
| Elastic sheet | Spring steel |
| Axles | Brass |
| Sleeves | Stainless steel |
| Linkages | Ductile resin |
